# Injury Risk in Pole Vaulters: The Impact of Landing Technique and Experience Level in an Observational Study of 1,012 Athletes and 6,751 Attempts

**DOI:** 10.7759/cureus.80036

**Published:** 2025-03-04

**Authors:** Jeremy Scott, Conner Howard, Christopher Hendrix, Andrea Siu, Jacqueline Nguyen, Spencer Chang, Leila Ben-Youssef

**Affiliations:** 1 Orthopaedic Surgery, Oklahoma State University Center for Health Sciences, Tulsa, USA; 2 Research Institute, Hawaii Pacific Health, Honolulu, USA; 3 Orthopaedic Surgery, San Mateo Medical Center, San Mateo, USA; 4 Orthopaedic Surgery, Pali Momi Medical Center, Aiea, USA

**Keywords:** landing injuries, orthopaedics, pole vault, sports, track and field

## Abstract

Background: Pole vaulting is a complex track and field event where athletes sprint down a runway, using a flexible pole to propel themselves over a horizontal crossbar. While safety regulations have reduced catastrophic injury rates, lower extremity injuries remain prevalent and demand further investigation. However, injury risks remain a concern due to the complexity of the sport and repetitive fall from heights. This study aims to investigate the correlation between landing mechanics and injury rates in pole vaulting. The authors hypothesize that improper landing techniques significantly increase the risk of injury, particularly in less experienced athletes.

Methods: This study was conducted at a national indoor pole vault event consisting of 1,012 athletes, ranging from beginner to elite level. Data was collected on skill level, landing location and position, and injuries. Only injuries sustained during a competitive vault attempt were considered for data analysis, which included descriptive statistics.

Results: The landing injury rate was low at 0.12%, with eight injuries occurring in 6,751 vault attempts. Notably, no injuries occurred in athletes with optimal landing mechanics. All reported injuries affected the lower extremities and were exclusively observed in less experienced vaulters.

Conclusion: Though pole vaulting is a relatively safe sport, catastrophic injuries are possible and the injuries that do occur are preventable, as this study reveals they occur at a higher rate in inexperienced vaulters as well as with an improper landing technique. Coaches and athletes should emphasize proper landing techniques to prevent injuries and improve performance.

## Introduction

Track and field is among the most popular sports in the United States with over four million participants per year [1]. Athletes range from early youth to elite-level athletes. The increasing number of specialized training centers and participation in national events suggests a growing interest in pole vaulting [2]. The pole vault is a track and field event that involves sprinting down a runway while using a long, flexible pole to project yourself over a horizontal crossbar. Current heights for elite athletes commonly exceed 19 feet (5.80 meters) for men and 16 feet (4.90 meters) for women. Speed must be prioritized to jump as high as possible while simultaneously ensuring safe landings. This presents unique and inherent injury risks comparable to extreme sports such as snowboarding, skiing, and skateboarding [3-5].

Lower extremity (LE) injuries are common among running and jumping events in track and field [6-9], with injury rates for collegiate pole vaulters reported at 8.65 per 1,000 athlete-exposures (AEs), while high school pole vaulters experience 7.1 injuries per 1,000 AEs [10]. A prospective study in 2015 found that 41% of collegiate pole vaulters sustained at least one injury in a single season, with 60% involving the LEs [11]. Similarly, injury rates among high school pole vaulters are approximately 7.1 per 1,000 AEs, with LE trauma accounting for 71.5% of injuries [11]. Incorrect landing patterns are the primary cause of both non-catastrophic and catastrophic injuries in pole vaulting [11]. Historically, pole vaulting has ranked among the sports with the highest rates of catastrophic injuries in high school and collegiate athletics [12,13]. Between 1982 and 1998, there were 32 catastrophic injuries, with 94% involving improper landings near the edge of the pad or in the vault box, resulting in 16 fatalities and six cases of incomplete neurological recovery. In 2002 alone, six catastrophic injuries led to three deaths [13,14].

In 2003, the National Collegiate Athletic Association (NCAA), National Federation of State High School Associations (NFHS), and USA Track and Field (USATF) implemented rule changes aimed at enhancing safety in pole vaulting [12,13]. Key changes included increasing landing pad dimensions and adding padding or removing hard surfaces around the pad. These adjustments have led to an 88% reduction in catastrophic injuries, with no fatalities reported since 2003 [12,13]. However, the average annual rate of catastrophic injuries from landing in the vault box area increased significantly in the years leading up to 2011-2012 [12,13]. Since then, additional safety measures, including the ASTM 2949 box collar modification, have been introduced. While the long-term impact of these interventions remains unclear, future studies should evaluate their effectiveness in reducing catastrophic injuries. Due to persistent concerns about landing-related injuries in pole vaulting, further evidence is needed on this issue. This study aimed to analyze the relationship between landing characteristics and injury rates in pole vaulting. The authors hypothesize that improper landing characteristics increase the risk of injury.

## Materials and methods

This observational study included pole vaulters competing at the 2015 National Pole Vault Summit, held indoors in Reno, Nevada. The event comprised 65 competitions using 12 runways and three landing pit models. Each competition level was defined based on athlete experience and eligibility criteria. The 'High School' category included athletes in grades 9-12 (or equivalent secondary school levels). 'College' referred to currently enrolled collegiate athletes, while 'Elite' consisted of professional and high-performance post-collegiate vaulters. 'Masters' included non-elite athletes over the age of 35, and the 'Open' category comprised post-collegiate competitors who did not meet the Elite or Masters classification. 'Beginner' status was self-selected by participants and typically included first- or second-year vaulters or those without prior competition experience. All pit models used at the event exceeded safety standards established by the NFHS, NCAA, and USATF, including specifications outlined in the 2015 NFHS Track and Field Pre-Meet Notes [15]. Medical coverage was provided by athletic trainers, a paramedic, a resident orthopedic surgeon, a resident emergency medicine physician, and a fellowship-trained sports medicine orthopedic surgeon.

The study received Institutional Review Board (IRB) approval, with a waiver of consent for non-injured participants (IRB#20151776). Injured participants provided informed consent before collecting additional information related to their injury. This methodology adheres to consensus guidelines for epidemiological studies in track and field [16].

Data collection

**Figure 1:**
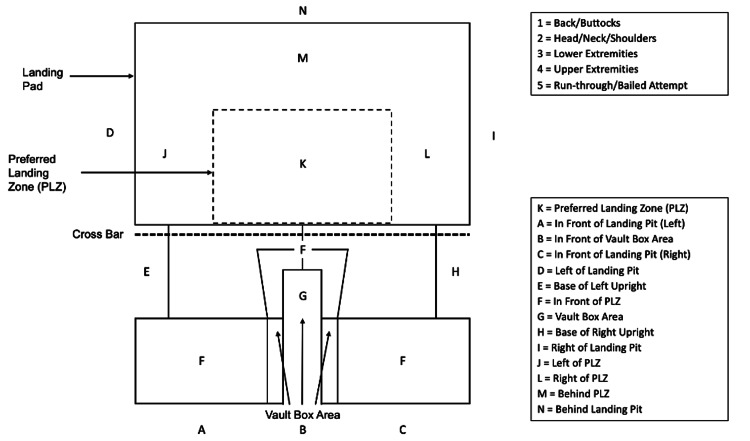
Pole Vault Landing Pit Illustration and Landing Position Descriptions

Demographic data were obtained from event registration records. Landing location and position for each vault attempt were recorded by 49 trained volunteers stationed at each pit. Volunteers received a brief orientation before the event, which included standardized instructions on identifying landing zones and positions. They were provided with a visual reference guide and a standardized data collection sheet to ensure consistency. Event officials were available to clarify questions and resolve discrepancies during data collection. Volunteers documented landing locations (labeled A-O) and landing positions (labeled 1-5) for each attempt (Figure 1).

Location referred to the initial point of contact on the landing pit, while position described the body part or anatomical region that made primary contact. Positions 1 through 4 were used for completed vaults, and Position 5 was designated for incomplete or bailed attempts. Optimal landing - defined as contact within the center of the pit, known as the preferred landing zone (PLZ), landing on the back or buttocks - was classified as K1.

Injury data included the mechanism of injury and clinical diagnoses made by on-site medical staff. Injuries were identified through two primary methods: self-reporting by athletes and direct observation by event spotters. Athletes reporting an injury were directed to an on-site medical station staffed by certified athletic trainers, an emergency medicine resident, an orthopedic surgery resident, and a fellowship-trained sports medicine orthopedic surgeon. If a spotter suspected an injury based on an abnormal landing or athlete distress, they alerted medical personnel for further assessment. Only injuries occurring during competition attempts and confirmed by medical staff were included in the analysis.

Data analysis

Descriptive statistics were generated for demographics, landing location and position, and injury data using Microsoft Excel (Microsoft Corp., Redmond, WA, USA). Landing location data were collected for 5,876 of the 6,751 (87.0%) competition attempts, while landing position data were available for 6,085 (90.1%) attempts. The discrepancy in data collection for location and position resulted from run-through attempts (n = 209).

Independent t-tests were conducted to compare best heights achieved between K1 and non-K1 landings across competition levels. Fisher’s exact test assessed differences in landing injuries between K1 and non-K1 landings. Statistical analyses were performed in Stata (StataCorp, College Station, TX, USA), with significance set at p < 0.05.

## Results

Participant demographics and attempt data

**Figure 2:**
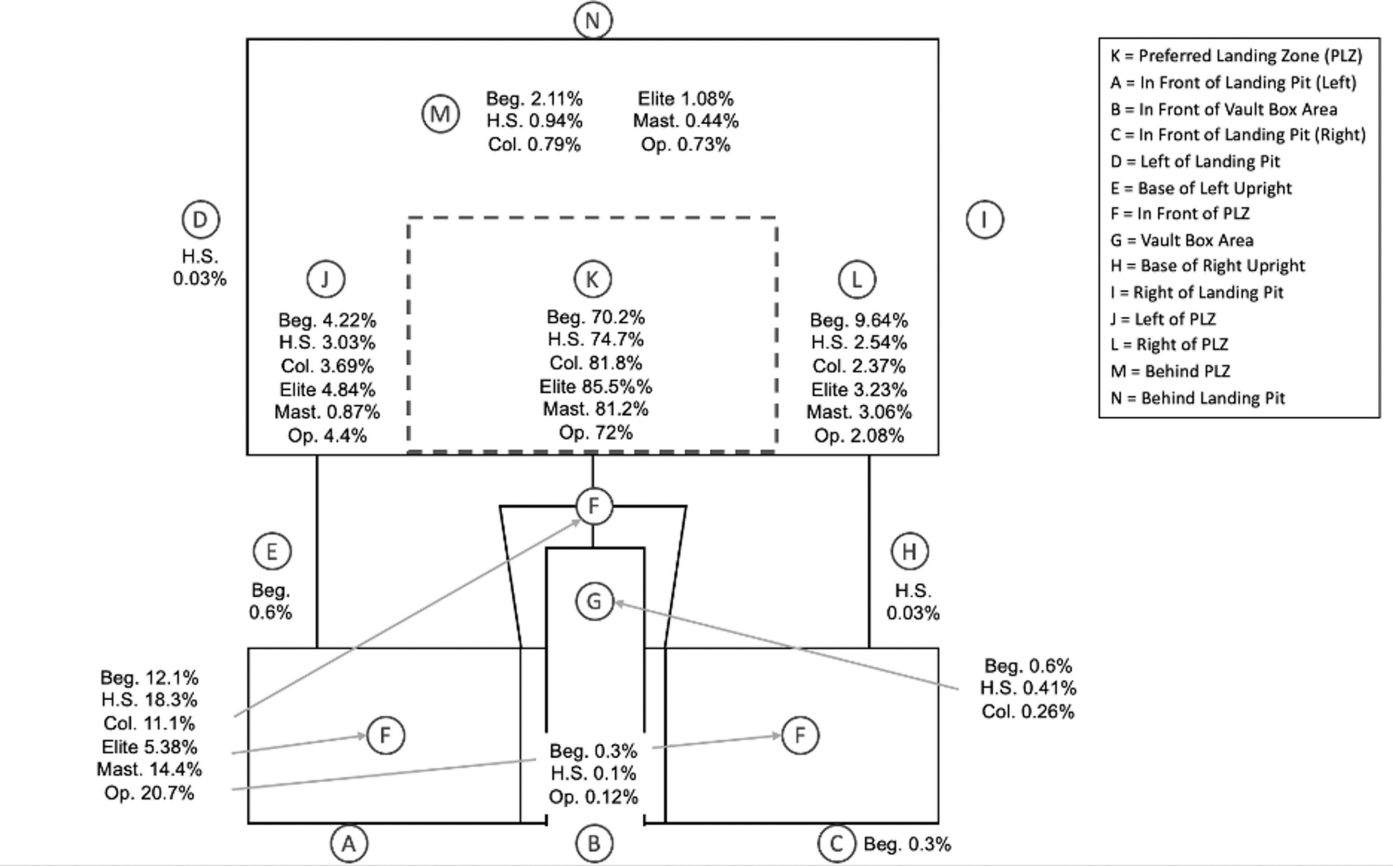
Landing Location Frequencies by Competition Level

A total of 1,012 pole vaulters participated in the event, accounting for 6,751 competition attempts (Table 1). The High School (261 males, 337 females) and Open (65 males, 49 females) groups were the largest, while the Masters group included only males (n = 49), no female athletes participated in this category as the event did not have a designated female Masters division. The Elite group had the fewest athletes (15 males, 10 females). The distribution of landing locations by competition level is shown in Figure 2.

**Table 1:** Participant Demographics and Competition Results *Values represented as mean ± standard deviation (range) in meters; no entries for female Masters division.

	All			Males			Females		
	Participants, No.	Attempts, No.	Best Height*	Participants, No.	Attempts, No.	Best Height*	Participants, No.	Attempts, No.	Best Height*
Beginners	46	340	2.09 ± 0.48 (1.22 - 3.04)	20	149	2.34 ± 0.52 (1.52 - 3.04)	26	191	1.89 ± 0.35 (1.22 - 2.44)
High School	675	4660	3.2 ± 0.71 (1.73 - 5.18)	311	2159	3.72 ± 0.61 (1.93 - 5.18	364	2501	2.80 ± 0.49 (1.73 - 4.06)
College	68	422	3.92 ± 0.71 (2.5 - 5.18)	35	200	4.49 ± 0.49 (3.6 - 5.3)	33	222	3.40 ± 0.42 (2.15 - 4.1)
Elite	28	193	4.99 ± 0.59 (4.1 - 5.92)	18	118	5.42 ± 0.27 (4.9 - 5.92)	10	75	4.34 ± 0.16 (4.1 - 4.6)
Masters	49	279	3.39 ± 0.57 (2.06 - 4.45)	49	279	3.39 ± 0.57 (2.06 - 4.45)	-	-	-
Open	146	857	3.9 ± 0.68 (2.4 - 5.35)	89	492	4.33 ± 0.46 (3.4 - 5.35)	57	365	3.33 ± 0.47 (2.4 - 4.15)
Total	1012	6751	3.34 ± 0.84 (1.22 - 5.92)	522	3397	3.82 ± 0.77 (1.52 - 5.92)	490	3354	2.89 ± 0.6 (1.22 - 4.6)

Across all competition levels, the PLZ, or "K" location, was the most frequent landing site, with landing rates ranging from 63.7% to 86.1% for males and 69.5% to 84.7% for females (Table 2). Thirty (0.44%) attempts missed the landing pit, with the highest miss rate observed among High School and Beginner males (1.1%). Additionally, 19 (0.28%) attempts ended in landings within the vault box area.

**Table 2:** Landing Location Frequencies by Competition Level (N = 5,876) *Total attempts recorded for landing location per competition level; Values represented as No. (%); No recordings for locations A, I, N. PLZ: preferred landing zone

Location	Competition Level (No.*)					
	Beginners (332)	High School (3,932)	College (379)	Elite (186)	Masters (229)	Open (818)
In Front of Vault Box Area (B)						
Males	0	3 (0.19%)	0	0	0	1 (0.21%)
Females	1 (0.54%)	1 (0.04%)	0	0	-	0
In Front of Landing Pit (C)						
Males	1 (0.68%)	0	0	0	0	0
Females	0	0	0	0	-	0
Left of Landing Pit (D)						
Males	0	0	0	0	0	0
Females	0	1 (0.04%)	0	0	-	0
Base of Left Upright (E)						
Males	1 (0.68%)	0	0	0	0	0
Females	1 (0.054%)	0	0	0	-	0
In Front of PLZ (F)						
Males	20 (13.7%)	265 (16.4%)	19 (10.7%)	6 (5.22%)	33 (14.4%)	84 (17.7%)
Females	20 (10.8%)	454 (19.6%)	23 (11.4%)	4 (5.63%)	-	85 (24.7%)
Vault Box Area (G)						
Males	2 (1.37%)	12 (0.74%)	1 (0.56%)	0	0	0
Females	0	4 (0.17%)	0	0	-	0
Base of Right Upright (H)						
Males	0	0	0	0	0	0
Females	0	1 (0.04%)	0	0	-	0
Left of PLZ (J)						
Males	14 (9.59%)	62 (3.83%)	12 (6.78%)	6 (5.22%)	2 (0.87%)	24 (5.06)
Females	0	57 (2.46%)	2 (0.99%)	3 (4.23%)	-	12 (3.49%)
PLZ (K)						
Males	93 (63.7%)	1,190 (73.6%)	139 (78.5%)	99 (86.1%)	186 (81.2%)	350 (73.8%)
Females	140 (75.3%)	1,745 (75.4%)	171 (84.7%)	60 (84.5%)	-	239 (69.5%)
Right of PLZ (L)						
Males	8 (5.48%)	60 (3.71%)	4 (2.26%)	2 (1.74%)	7 (3.06%)	9 (1.90%)
Females	24 (12.9%)	40 (1.73%)	5 (2.48%)	4 (5.63%)	-	8 (2.33%)
Behind PLZ (M)						
Males	7 (4.79%)	26 (1.61%)	2 (1.13%)	2 (1.74%)	1 (0.44%)	6 (1.27%)
Females	0	11 (0.48%)	1 (0.50%)	0	-	0

Landing position analysis

**Figure 3:**
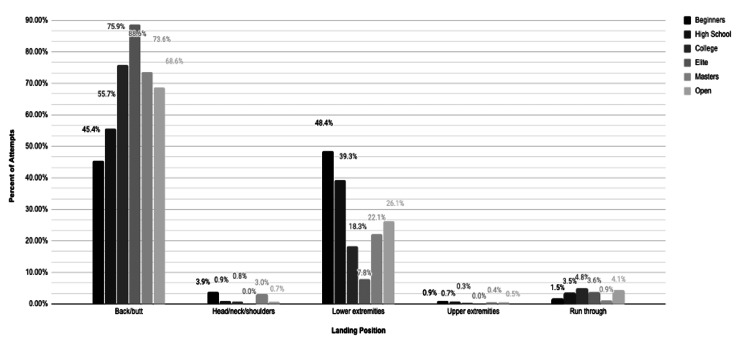
Landing Position Frequencies by Competition Level

The most common landing position across all groups was on the back/buttocks (Figure 3).

Among males, back/buttocks landings accounted for 57.7% to 82.7% of attempts, while females’ most common landing position varied by competition level (Table 3). Lower extremity landings were most frequently observed among Beginner (62.8%) and High School (50.2%) females. Female vaulters in the College, Elite, and Open groups landed primarily on their back/buttocks, with rates ranging from 55.9% to 86.7%. Overall, 51.5% of landings met the optimal K1 criteria (center pit, back/buttocks).

**Table 3:** Landing Position Frequencies by Competition Level (N = 6,085) *Total attempts recorded for landing location per competition level; Values represented as No. (%); No recordings for locations A, I, N. PLZ: preferred landing zone

Position	Competition Level (No.*)					
	Beginners (337)	High School (4,073)	College (398)	Elite (193)	Masters (231)	Open (853)
Back/Buttocks (1)						
Males	86 (57.7%)	1,184 (70.1%)	153 (82.7%)	106 (89.8%)	170 (73.6%)	78.1%)
Females	67 (35.6%)	1,085 (45.5%)	149 (70.0%)	65 (86.7%)	-	204 (55.9%)
Head/Neck/Shoulders (2)						
Males	13 (8.72%)	20 (1.18%)	1 (0.54%)	0	7 (3.03%)	6 (1.23%)
Females	0	16 (0.67%)	2 (0.94%)	0	-	0
Lower Extremities (3)						
Males	45 (30.2%)	403 (23.9%)	22 (11.9%)	9 (7.63%)	51 (22.1%)	86 (17.6%)
Females	118 (62.8%)	1,197 (50.2%)	51 (24.0%)	6 (8.0%)	-	137 (37.5%)
Upper Extremities (4)						
Males	2 (1.34%)	12 (0.71%)	1 (0.54%)	0	1 (0.43%)	1 (0.20%)
Females	1 (0.53%)	15 (0.63%)	0	0	-	3 (0.82%)
Run-through (5)						
Males	3 (2.01%)	70 (4.14%)	8 (4.32%)	3 (2.54%)	2 (0.87%)	14 (2.87%)
Females	2 (1.06%)	71 (2.98%)	11 (5.16%)	4 (5.33%)	-	21 (5.75%)

Elite vaulters achieved K1 landings most often (82.8%), while Beginners were the least likely to achieve K1 (28.0%). Athletes with K1 landings achieved significantly higher best heights than those with non-K1 landings (p < 0.001) (Table 4, Figure 3)

**Table 4:** K1 vs. Non-K1 Landings by Competition Level *Statistically significant difference between K1 and non-K1 landings for best heights (p < 0.001) and landing injuries (p=0.003).

	All*		Males		Females	
Competition Level	K1, No. (%)	Non-K1, No. (%)	K1, No. (%)	Non-K1, No. (%)	K1, No. (%)	Non-K1, No. (%)
Beginners	93 (28.0%)	239 (72.0%)	56 (38.4%)	90 (61.6%)	37 (19.9%)	149 (80.1%)
High School	1,868 (47.5%)	2,064 (52.5%)	965 (59.7%)	653 (40.4%)	903 (39.0%)	1,411 (61.0%)
College	267 (70.5%)	112 (29.6%)	128 (72.3%)	49 (27.7%)	139 (68.8%)	63 (31.2%)
Elite	154 (82.8%)	32 (17.2%)	96 (83.5%)	19 (16.5%)	58 (81.7%)	13 (18.3%)
Masters	159 (69.4%)	70 (30.6%)	159 (69.4%)	70 (30.6%)	-	-
Open	484 (59.2%)	334 (40.8%)	310 (65.4%)	164 (34.6%)	174 (50.6%)	170 (49.4%)
Total	3,025 (51.5%)	2,851 (48.5%)	1,714 (62.1%)	1,045 (37.9%)	1,311 (42.1%)	1,806 (57.9%)

Injury incidence and types

A total of 14 injuries were reported during competition, yielding an overall injury rate of 0.21% (2.07 injuries per 1,000 athlete-exposures). Eight of these 14 injuries (57.1%) were related to landing, resulting in a landing-specific injury rate of 0.12% (1.18 per 1,000 athlete-exposures) (Table 5). Landing injuries occurred at a much higher frequency outside of the K1 criteria, with a statistically significant difference between K1 and non-K1 landings (p = 0.003). Injury locations included F (in front of PLZ), G (vault box area), and K (PLZ). Only one injury occurred with a back/buttocks landing, specifically in the vault box area.

**Table 5:** Landing Injury Rates by Competition Level Injury rate calculated out of 6,751 total attempts. AE: athlete-exposures

Competition Level	All			Males			Females		
	Injuries, No.	Injury Rate	Incidence per 1,000 AEs	Injuries, No.	Injury Rate	Incidence per 1,000 AEs	Injuries, No.	Injury Rate	Incidence per 1,000 AEs
Beginners	1	0.29%	2.94	0	0	0	1	0.52%	5.24
High School	6	0.13%	1.29	3	0.14%	1.39	3	0.12%	1.2
College	1	0.24%	2.37	1	0.50%	5	0	0	0
Elite	0	0	0	0	0	0	0	0	0
Masters	0	0	0	0	0	0	0	0	0
Open	0	0	0	0	0	0	0	0	0
Total	8	0.12%	1.18	4	0.12%	1.18	4	0.12%	1.18

Injury characteristics by competition level

High School athletes accounted for six of the eight landing injuries (three males, three females), with a landing injury rate of 0.13% (1.29 per 1,000 athlete-exposures). The highest landing injury rates were observed in the Beginner (0.29%; 2.94 per 1,000 athlete-exposures) and College (0.24%; 2.37 per 1,000 athlete-exposures) groups. No landing injuries occurred among Elite, Masters, or Open-level competitors.

Injury mechanism and type

Four of the eight landing injuries (50%) affected the lower extremities, with feet-first landings accounting for three of these four injuries (75%). Ligament sprains were the most common injury type, comprising 37.5% (n = 3) of landing injuries (Table 6).

**Table 6:** Descriptions and Characteristics of Landing Injuries *Diagnosis based on clinical findings (physical exam) during on-site evaluations. PLZ: preferred landing zone; LE: lower extremity; UE: upper extremity; HNS: head, neck, shoulders; ACL: anterior cruciate ligament; UCL: ulnar collateral ligament

Competition Level	Sex	Landing Pattern (location; position)	Landing Description	Diagnosis*
Beginner	F	K3 (PLZ; LE)	Feet-first with knee hyperextended	Knee (ACL) Sprain
High School	F	F3 (In front of PLZ; LE)	Feet-first on pronated foot	Lateral Ankle Sprain
High School	F	F3 (In front of PLZ; LE)	Feet-first on pronated foot	Lateral Ankle Sprain
High School	F	K4 (PLZ; UE)	On hand with thumb abducted and hyperextended	Thumb (UCL) Sprain
High School	M	K4 (PLZ; UE)	On adducted arm	Shoulder Contusion
College	M	K2 (PLZ; HNS)	On head, neck, shoulder due to over-rotation	Neck/Upper Back Strain
High School	M	G2 (Box area; HNS)	On head, neck, shoulder at conjunction of pit and vault box area	Concussion, Neck/Back Strain and Contusion
High School	M	G1 (Box area; back and buttocks)	On back in plant box area	Low Back/Gluteal Contusion

## Discussion

This study aimed to analyze the relationship between landing characteristics and injury rates in pole vaulting, with particular focus on the role of landing patterns in injury prevention. Our findings support the hypothesis that improper landing characteristics are associated with increased injury risk. The majority of injuries in this study were associated with non-K1 (improper) landings, highlighting the importance of proper technique in minimizing injury risk, and there was a statistically significant difference in injury rates between K1 and non-K1 landings. This emphasizes the importance of correct landing techniques in lowering injury risks during pole vaulting.

In alignment with prior research, our study identified LE impact as a common mechanism of injury, with ligament sprains being the most frequent injury type [11]. LE injuries in this study predominantly occurred due to feet-first landings, supporting findings in previous literature [11]. However, it is important to note that all landing pits at this event were essentially brand new, which may have reduced the incidence of feet-first landings compared to real-world conditions where older, more compacted pits might increase injury risk. High school athletes were the most frequently injured group, although this cohort also accounted for the largest number of attempts. Interestingly, injury rates observed in this study (1.29 per 1,000 AEs for high school athletes; 2.37 per 1,000 AEs for collegiate athletes) were markedly lower than those reported in earlier studies (7.1 per 1,000 AEs for high school and 6.5-7.9 per 1,000 AEs for collegiate athletes) [10,11]. The lower injury rates observed in this study may reflect advancements in safety equipment, improved coaching practices, and increased athlete awareness compared to earlier studies. Additionally, the presence of standardized pit conditions and on-site medical staff may have contributed to more immediate injury management, potentially reducing both the occurrence and severity of injuries. The overall injury rate observed in this study (2.07 per 1,000 AEs) is markedly lower than previously reported rates among high school (7.1 per 1,000 AEs) and collegiate pole vaulters (7.9 per 1,000 AEs) [11,17]. This discrepancy may be attributed to improvements in landing surface technology, increased safety awareness, or differences in reporting thresholds across studies. Additionally, the presence of experienced medical personnel at the event may have contributed to more immediate injury management, potentially reducing the severity of injuries and subsequent reporting patterns [11,17].

Contrary to previous findings linking greater experience and higher vault heights with increased injury risk [11], our data indicated that beginner-level athletes had the highest landing injury rate (2.94 per 1,000 AEs). Moreover, no landing injuries occurred among competitors in the Elite, Masters, or Open categories, which were generally associated with greater experience and higher achieved heights. Collectively, these results suggest that less experienced athletes may be more prone to unsafe landings, potentially due to limited technical knowledge and control.

A study by Boden et al. noted that catastrophic injuries, especially those involving the vault box area, tend to occur more frequently at the college and professional levels [13]. However, in our observational study, no catastrophic injuries were sustained, despite 30 (0.44%) attempts missing the landing pit and 19 (0.28%) landings in the vault box area. This aligns with recent evidence suggesting that ongoing safety measures in pole vaulting, such as enhanced padding and improved equipment, may effectively reduce the risk of severe injuries. The absence of catastrophic injuries in our study highlights the potential efficacy of these preventative measures in minimizing life-threatening incidents [10].

A proper landing (K1) involves landing on the back in the center of the landing pit, allowing force distribution over a safe, padded area [18]. Force dissipation is calculated as 𝐹𝑑 = 𝑛 × 𝑣 × 𝐴 where 𝑛 is the material’s damping effect, 𝑣 is velocity, and A is surface area. In pole vault landing systems, 𝑛 typically ranges from 0.2 to 0.5, depending on the polyurethane foam used by the manufacturer [19]. As athletes jump higher, landing velocities increase due to gravity’s prolonged effect. Studies on falls from height show that increasing the fall from 30 cm to 75 cm increases vertical ground reaction forces from 3.31 to 5.68 times the athlete’s body weight [20]. While biomechanical analysis of pole vault falls could provide valuable data on injury risks, such studies are impractical due to athlete safety concerns [20]. Given the risk of injury, maximizing landing surface area is crucial. The back, accounting for 18% of total body surface area, provides about 324 cm² of contact area in a proper K1 landing, which is significantly larger than the surface area when landing on one or two feet. This larger contact area plays a critical role in force dissipation, as it allows for better distribution of impact forces across a broader surface, reducing the risk of injury. Additionally, the back’s ability to effectively dampen impact forces helps absorb and dissipate energy, minimizing the concentration of force on any single point, which is crucial for injury prevention. In support of this, no injuries occurred following back-first (K1) landings in our study. Beginner and high school vaulters demonstrated the highest rates of non-K1 landings, with frequent occurrences of feet-first landings, particularly among female athletes. All knee and ankle injuries occurred in the beginner and high school groups and were associated with feet-first landings. Additionally, all upper extremity and head/trunk injuries were linked to direct impact with the respective body regions and/or landing outside the padded zones. These findings emphasize the importance of teaching safe landing techniques early in a vaulter’s training.

Two potential explanations for the higher rates of improper landings among novice vaulters are proposed. First, inexperience or inadequate coaching may contribute to unsafe landings. Without early emphasis on proper landing techniques, beginner athletes may lack the knowledge or practice needed to land safely. As demonstrated in other sports, such as martial arts, early instruction on safe landing techniques can be essential for injury prevention [18]. In pole vaulting, variables such as pole stiffness, bend, grip height, horizontal speed, and take-off direction can affect landing characteristics [21-24]. For less experienced athletes, these variables may contribute to increased landing variability and risk.

Second, the biomechanical aspects of pole vaulting may predispose beginner athletes to horizontal trajectories and feet-first landings due to lower speeds and heights [22,23]. Although back-first landings should be encouraged, athletes must avoid "sticking" landings to prevent excessive impact on the feet, especially in spiked shoes, which can increase the risk of ankle injuries.

This study has several limitations. Landing characteristics were recorded by event volunteers with varying experience levels, introducing potential observer bias and inconsistencies. Unlike studies using video review, this real-time observation method may have led to underreporting of certain landing patterns, such as neck and shoulder impacts.

Injury data were limited to cases reported to medical staff, meaning minor injuries may have been missed. While athletes primarily self-reported injuries, spotters flagged abnormal landings for evaluation, but the lack of standardized post-competition tracking may have contributed to underreporting.

Additionally, this study was conducted in 2015, and improvements in training, safety equipment, and injury prevention may have altered injury patterns since then. The findings may not fully reflect current practices, highlighting the need for updated research.

Another limitation is the absence of long-term follow-up, as this study assessed injuries only during a single competition. The small number of injuries limits generalizability but reinforces a key finding: pole vaulting remains relatively safe with proper landing mechanics, whereas deviations in technique increase injury risk.

The findings of this study highlight the importance of understanding tissue responses to mechanical stress, particularly in young athletes undergoing rapid growth and musculoskeletal development. Improper landing techniques may not only increase the risk of acute injuries but could also contribute to chronic adaptations, such as stress-related changes in musculoskeletal tissues. Repeated biomechanical stressors have been associated with the formation of lamellar bone in non-osseous tissues, including muscles, subcutaneous tissue, and periarticular structures. This process, observed in periarticular muscles near the hip, elbow, knee, and shoulder, may have implications for long-term athlete health and injury prevention strategies [25]. By further investigating these mechanisms, clinicians and sports scientists can develop more effective training protocols and safety interventions to minimize injury risk and optimize athlete longevity

Future research should address these gaps by incorporating long-term follow-up, biomechanical assessments, and a more comprehensive consideration of injury types, including minor and subclinical injuries. This would provide a more rigorous examination of the factors influencing landing patterns and safety in pole vaulting.

## Conclusions

The findings of this study highlight the importance of proper landing techniques in preventing injuries in pole vaulting. The higher frequency of improper landings among beginner and high school athletes emphasizes the need for early and structured training in safe landing mechanics. Future research should focus on biomechanical analyses of landing techniques to identify key risk factors and optimize training interventions. Additionally, the development of targeted training programs for beginner athletes, incorporating video feedback and structured drills, may help reduce injury risk and improve technique consistency. Expanding safety measures, such as improved pit design and real-time monitoring of landing patterns, could further enhance athlete protection and reduce injury incidence.
